# Deep learning for detection of Fuchs endothelial dystrophy from widefield specular microscopy imaging: a pilot study

**DOI:** 10.1186/s40662-024-00378-1

**Published:** 2024-03-18

**Authors:** Valencia Hui Xian Foo, Gilbert Y. S. Lim, Yu-Chi Liu, Hon Shing Ong, Evan Wong, Stacy Chan, Jipson Wong, Jodhbir S. Mehta, Daniel S. W. Ting, Marcus Ang

**Affiliations:** 1 Singapore National Eye Centre, 11 Third Hospital Avenue, Singapore, 168751 Singapore; 2 Singapore Eye Research Institute, Singapore, Singapore; 3 Duke-NUS Medical School, Ophthalmology and Visual Science Academic Clinical Research Program, Singapore, Singapore

**Keywords:** Deep learning, Cornea, Endothelium, Artificial intelligence

## Abstract

**Background:**

To describe the diagnostic performance of a deep learning (DL) algorithm in detecting Fuchs endothelial corneal dystrophy (FECD) based on specular microscopy (SM) and to reliably detect widefield peripheral SM images with an endothelial cell density (ECD) > 1000 cells/mm^2^.

**Methods:**

Five hundred and forty-seven subjects had SM imaging performed for the central cornea endothelium. One hundred and seventy-three images had FECD, while 602 images had other diagnoses. Using fivefold cross-validation on the dataset containing 775 central SM images combined with ECD, coefficient of variation (CV) and hexagonal endothelial cell ratio (HEX), the first DL model was trained to discriminate FECD from other images and was further tested on an external set of 180 images. In eyes with FECD, a separate DL model was trained with 753 central/paracentral SM images to detect SM with ECD > 1000 cells/mm^2^ and tested on 557 peripheral SM images. Area under curve (AUC), sensitivity and specificity were evaluated.

**Results:**

The first model achieved an AUC of 0.96 with 0.91 sensitivity and 0.91 specificity in detecting FECD from other images. With an external validation set, the model achieved an AUC of 0.77, with a sensitivity of 0.69 and specificity of 0.68 in differentiating FECD from other diagnoses. The second model achieved an AUC of 0.88 with 0.79 sensitivity and 0.78 specificity in detecting peripheral SM images with ECD > 1000 cells/mm^2^.

**Conclusions:**

Our pilot study developed a DL model that could reliably detect FECD from other SM images and identify widefield SM images with ECD > 1000 cells/mm^2^ in eyes with FECD. This could be the foundation for future DL models to track progression of eyes with FECD and identify candidates suitable for therapies such as Descemet stripping only.

## Background

Fuchs corneal endothelial dystrophy (FECD) is the most common posterior corneal dystrophy and one of the leading indications for corneal transplantation worldwide. In early FECD, guttae formation and endothelial cell density (ECD) decline manifest in the central cornea [1]. In advanced FECD, peripheral ECD highly correlates with disease severity [2]. Corneal transplantation techniques have allowed for more selective keratoplasty, and the replacement of only the diseased layers of the cornea [3]. However, current endothelial keratoplasty techniques still carry the possibility of graft rejection [4], and there remains a worldwide shortage of donor corneas for transplants [5]. Recent studies have suggested a role for Descemet stripping only (DSO), which involves creating a small 4 to 5 mm central descemetorhexis to remove diseased endothelium and guttae without the placement of any donor graft [6]. This is based on the principle that the central endothelium of FECD eyes is capable of self-regeneration via centripetal migration of healthy peripheral corneal endothelial cells that allows for spontaneous resolution of corneal edema [7].

Specular microscopy (SM) is a non-contact, non-invasive technique that sends light towards the cornea at an incidence angle and captures the reflected light from the interface between the endothelium and the aqueous humour [8]. Based on these images, in-built software automatically derives the ECD, the hexagonal endothelial cell ratio (HEX), and coefficient of variation (CV). The three parameters incorporated include ECD, CV, and HEX [8, 9].

Of recent, multiple case series of DSO with careful FECD patient selection have reported excellent success, with corneal edema clearing in up to 100% of patients [10]. The ideal patient for DSO would include those with peripheral ECD of > 1000 cells/mm^2^ without guttae [6]. This observational evidence demonstrated a potential that a machine learning approach could be trained in the identification of mild to moderate FECD eyes with healthy peripheral corneal endothelial reserves that would be useful to clinicians in selecting appropriate eyes that could benefit from this therapy. This deep learning (DL) model could potentially facilitate earlier intervention strategies in this subset of FECD eyes.

Thus far, no other studies have reported on the use of DL models for the above purpose. Earlier studies so far have developed DL systems for segmenting corneal endothelial cells and deriving the endothelial morphometric parameters, using either SM [11–22] or in vivo confocal microscopy [16, 23]. Artificial intelligence (AI) has also been shown to aid in detecting or predicting corneal disease progression [24], such as via anterior segment optical coherence tomography (AS-OCT) imaging [25]. Other DL models have evaluated ultrathin Descemet stripping automated endothelial keratoplasty (DSAEK) grafts with SM images [26], detected Descemet membrane endothelial keratoplasty (DMEK) graft detachments [27], and predicted the need for graft re-bubbling [28]. The primary aim of this novel study was hence to first assess the performance of a DL algorithm not only for the detection of FECD eyes from central SM images as a foundational step, but also to identify widefield SM images with ECD > 1000 cells/mm^2^ in eyes with FECD.

## Methods

### Study population and datasets

**Fig. 1:**
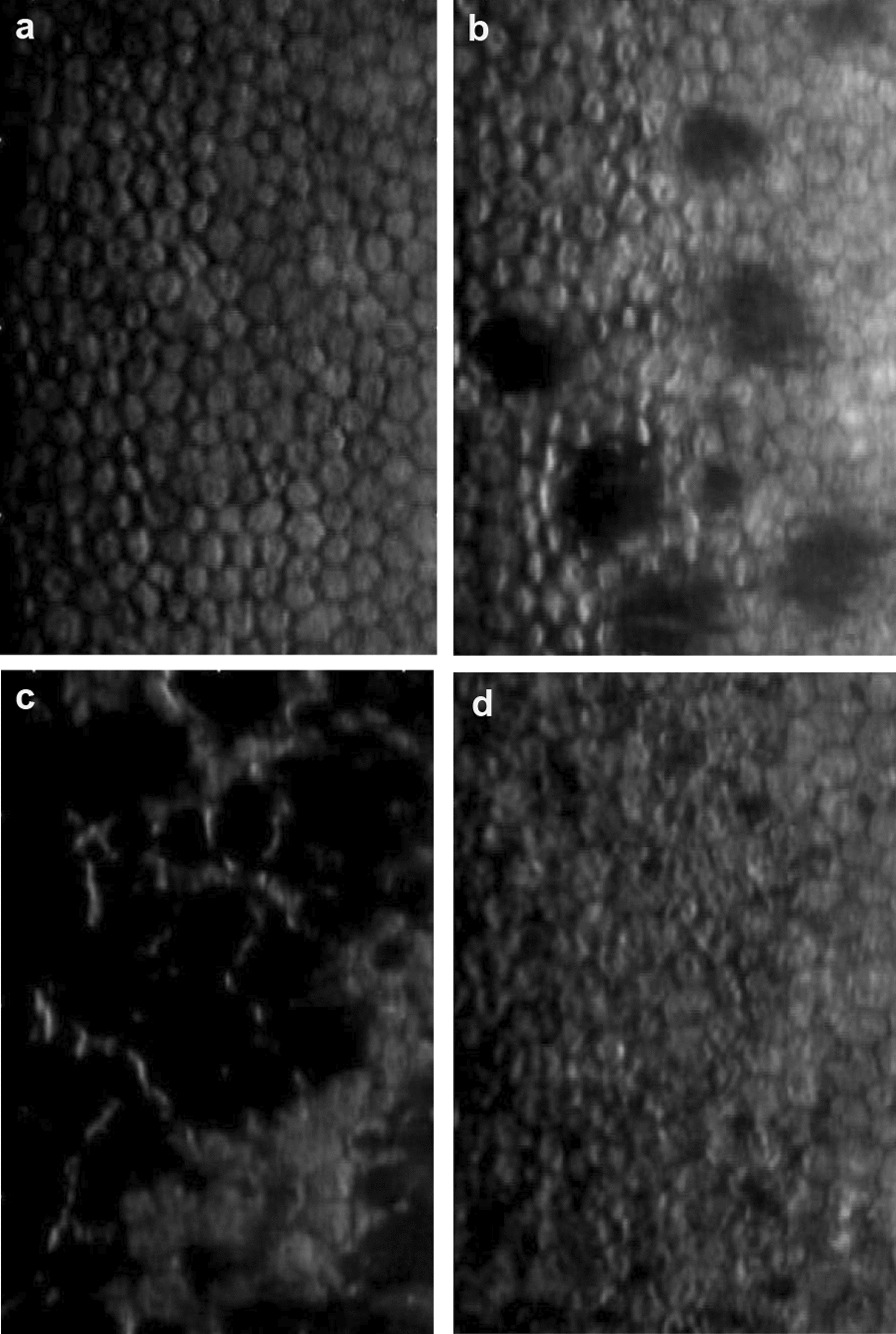
Various specular microscopy (SM) images that are used to train deep learning (DL) model 1. **a** Normal SM image. **b** SM image with Fuchs endothelial corneal dystrophy (FECD) (non-confluent guttae). **c** SM image with FECD (confluent guttae). **d** SM image with pigments on endothelium (uveitis)

We used de-identified high-resolution SM images to develop and evaluate the DL models. Ethics review and institutional board exemption were obtained from the SingHealth Institutional Review Board (IRB number 2018/2008). For the first DL model, 775 central SM images of the corneal endothelium were captured with a noncontact specular microscope Konan NSP-9900 (Konan Medical, Inc. Hyogo, Japan), and the SM parameters were calculated with the manual Center Method [16]. Images were diagnosed by trained ophthalmologists to classify 369 normal SM images (Fig. 1a), 173 SM images with FECD (Fig. 1b and c), and 233 with abnormal SM images due to other conditions e.g., pigments, iatrogenic endothelial damage, or uveitis, and etc. (Fig. 1c). We excluded images of poor quality from corneal edema or image artefacts due to eye movement or blinking, eyes with previous corneal surgery such as keratoplasty. A second independent dataset of central SM images from patients with a similar proportion of normal, FECD and other abnormal images was used for external validation for the first DL model, consisting of 90 subjects (180 eyes) graded by a trained cornea specialist (E.W.).

A third independent database consisting 753 paracentral and 557 peripheral SM images from only eyes with FECD, using a non-contact widefield SM system (CEM-530, Nidek Co., Ltd, Japan) was used to train the second DL model. The widefield SM is only available under Nidek and not Konan, and allows for an even larger viewing area, which captures an additional eight different paracentral images for every 1.5 clock hours 5 degrees from the center (radius of 0.6 mm from the centre) and six peripheral images for every 2 clock hours 27 degrees from the center (radius of 3.7 mm from the centre) of the cornea endothelium, giving a total of 15 SM images per eye. The imaging point was controlled by patient fixation and based on the patient’s primary line of sight [16].

### Outcomes

The aim of the first DL model was to differentiate between FECD and non-FECD eyes. Normal SM parameters were defined as having all three criteria of ECD of ≥ 2000 cells/mm^2^ [9], CV < 40% [10] and HEX > 60% [10]. FECD was diagnosed by trained ophthalmologists as having bilateral central guttae on slit-lamp examination, with an ECD < 2000 cells/mm^2^ or if ECD ≥ 2000 cells/mm^2^, then with HEX < 60% and/or CV > 40%. Eyes with mild to moderate FECD (defined by the Krachmer scale [29] of Grade 1 to 5 without corneal edema) were included in the study. Non-FECD with abnormal SM images were defined as having ECD < 2000 cells/mm^2^ due to other pathologies such as other endothelial dystrophies, previous intraocular surgeries, or anterior uveitis. Both the endothelial parameters of ECD, CV and HEX as well as SM images were incorporated into the training dataset for the first DL model.

The aim of the second DL model was to identify widefield SM images with ECD > 1000 cells/mm^2^ in eyes with FECD. The theoretical cut-off of peripheral ECD ≤ 1000 cells/mm^2^ is the exclusion criteria for DSO adapted from Moloney et al. [6], although not yet clinically validated. Only SM images without their clinical parameters were used for the training dataset in this second DL.

### Algorithm development

**Fig. 2:**
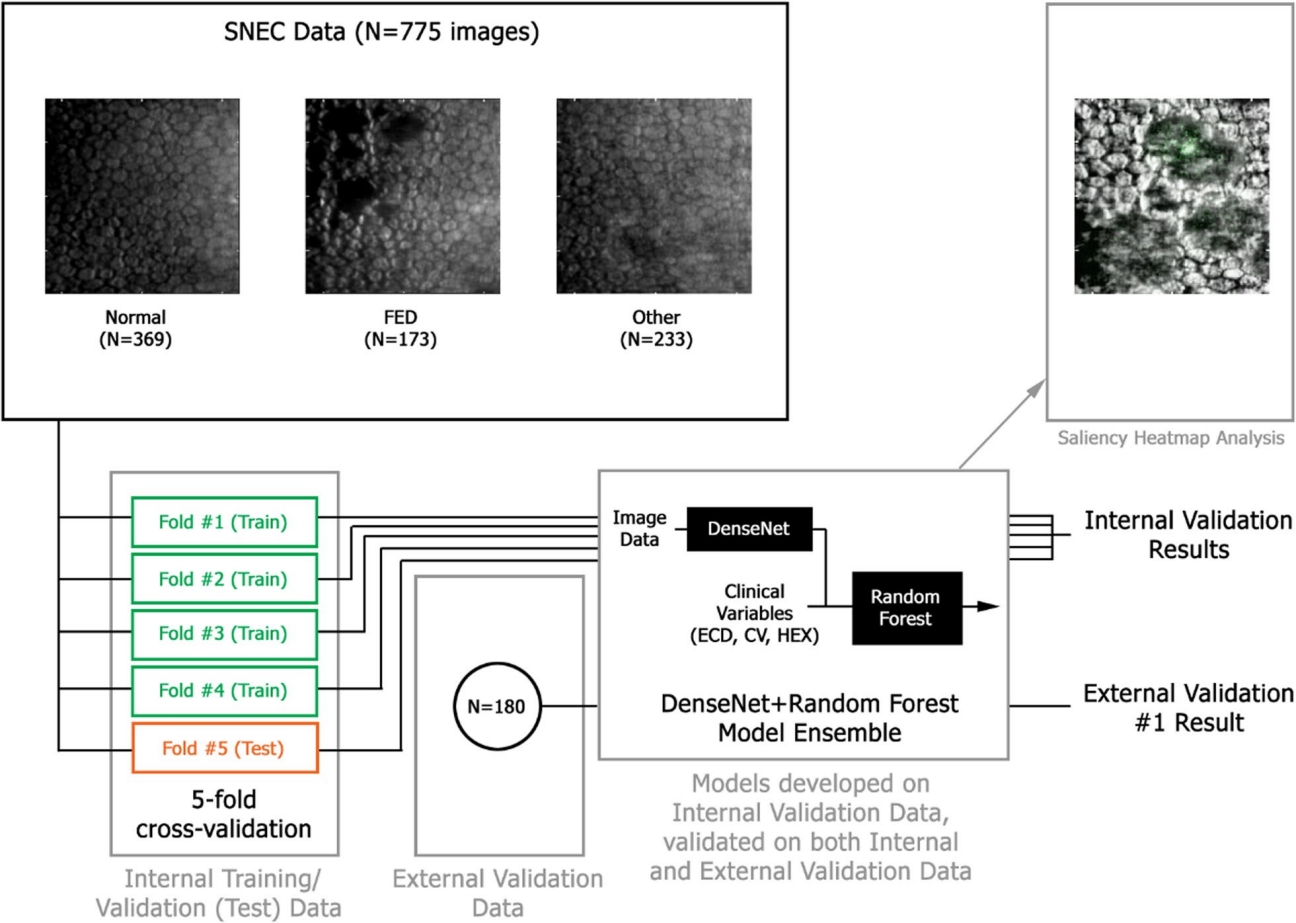
Flowchart demonstrating the training and cross-validation of the DenseNet-121 model for the first deep learning model. ECD, endothelial cell density; CV, coefficient of variation; HEX, hexagonal endothelial cell ratio

For the first DL model, for image-based classification of the SM images, a pre-trained DenseNet-121 architecture was used with fivefold cross-validation. DenseNet-121 was pre-trained with the ImageNet dataset. Then, for each of the cross-validation folds, the four folds assigned for training were further randomly divided into training data and internal validation data, in an approximately 4:1 ratio. The training data is then used to optimize the DenseNet model neuron weights with an initial learning weight of 0.001 and Nesterov momentum of 0.9, until accuracy converges on the internal validation data. The DenseNet model image-based predictions can then be obtained on the held-out test data, numerical outputs from the DenseNet model, for each image. The six values are then used as the inputs to a Random Forest classifier. The same cross-validation folds, as used for DenseNet training and validation, are retained. Hyperparameters relating to the number of tree estimators, the number of features to consider when looking for the best split, the maximum depth of the tree estimators and the quality criterion used are then optimized by grid search on the internal validation data for each fold. Again, the model with the best internal validation accuracy is used to evaluate the test data for each fold (Fig. 2).

**Fig. 3:**
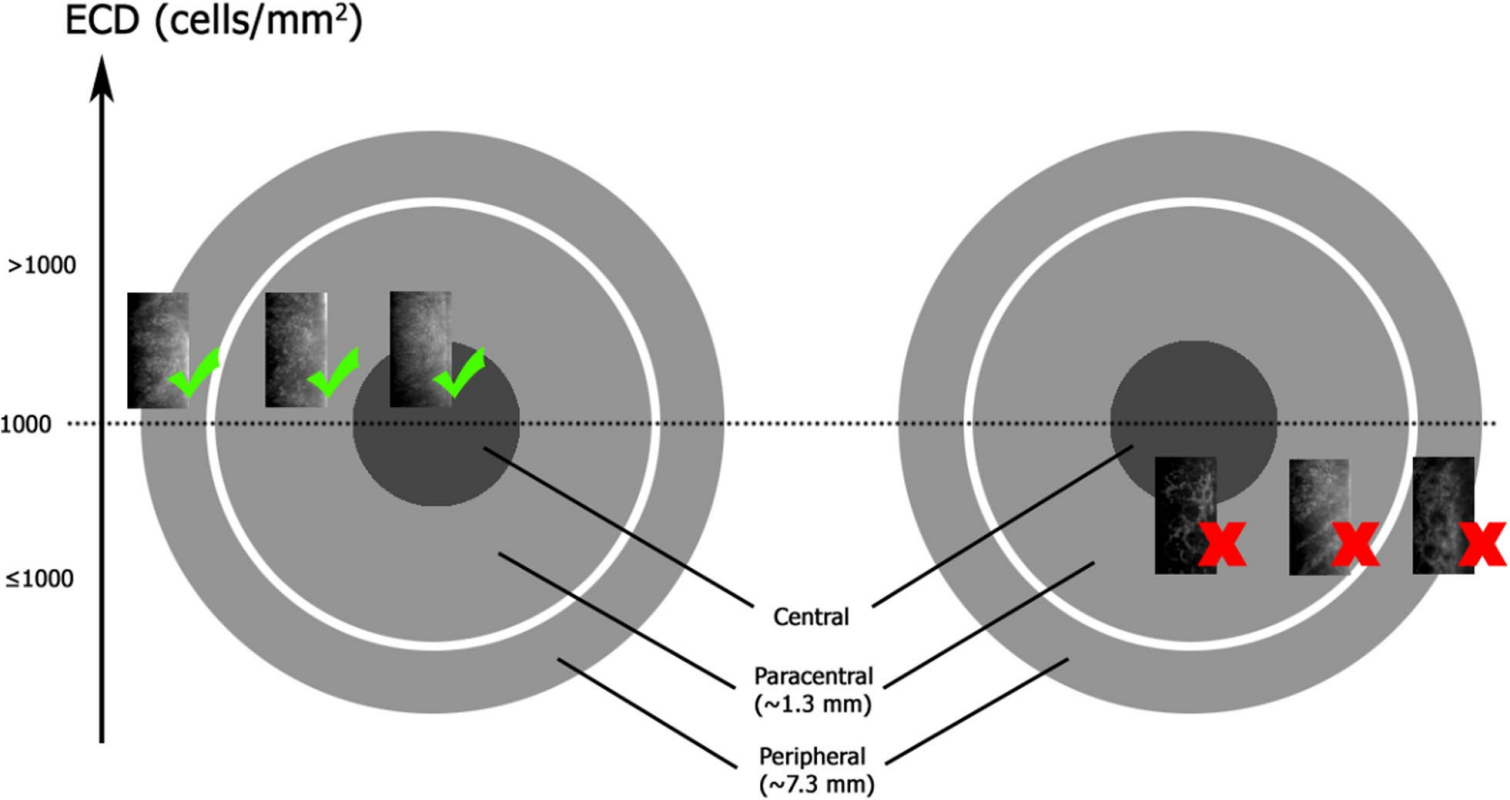
Figure demonstrating the clinical utility of the second deep learning model. Green ticks represent widefield specular microscopy (SM) images which have ECD > 1000 cells/mm^2^*.* Red crosses represent widefield SM images which have ECD ≤ 1000 cells/mm^2^

A second DenseNet-121 model was trained for the subsequent DL model. A new independent training set comprised of the paracentral scans (211 images with ECD ≤ 1000 cells/mm^2^, 571 images with ECD > 1000 cells/mm^2^), and the test set comprised of the peripheral scans (109 images with ECD ≤ 1000 cells/mm^2^, 478 images with ECD > 1000 cells/mm^2^) (Fig. 3). As with the previous DenseNet model, the training set was randomly divided into training and internal validation in a 4:1 ratio, and the same initial model parameters were used.

### Saliency maps

To understand which areas of the SM images were most likely used by the algorithm for the distinction between FECD and other non-FECD SM images, we generated saliency maps using Integrated Gradients, highlighting the areas in the image which contributed more towards the output (i.e., higher density of green pixels indicating a greater contribution).

### Statistical analysis

Statistical analyses were performed using SPSS software version 26.0 (SPSS, Chicago, IL, USA), and MATLAB 2019b (MathWorks, Natick, MA). We calculated the area under the curve (AUC), sensitivity and specificity. The 95% confidence intervals (CIs) for these performance metrics were estimated using the cross-validated sensitivity and specificity, on the full datasets.

## Results

We developed the first DL model using 775 SM images. We further validated the performance of the algorithm on the entire internal dataset through five-fold cross-validation, and on 180 SM images from the external test set.

First, we examined the performance of the algorithm for discrimination of abnormal (both FECD and non-FECD) from normal SM images. In the internal validation set, the AUC for detection of abnormal SM images was 0.92 (95% CI: 0.86–0.91), with a sensitivity of 0.86 (95% CI: 0.82–0.89) and specificity of 0.86 (95% CI: 0.84–0.91). In the external validation set, the AUC for discrimination of abnormal SM images was 0.82 (95% CI: 0.89–0.93) with a sensitivity of 0.74 (95% CI: 0.68–0.80) and specificity of 0.74 (95% CI: 0.68–0.80).

**Fig. 4:**
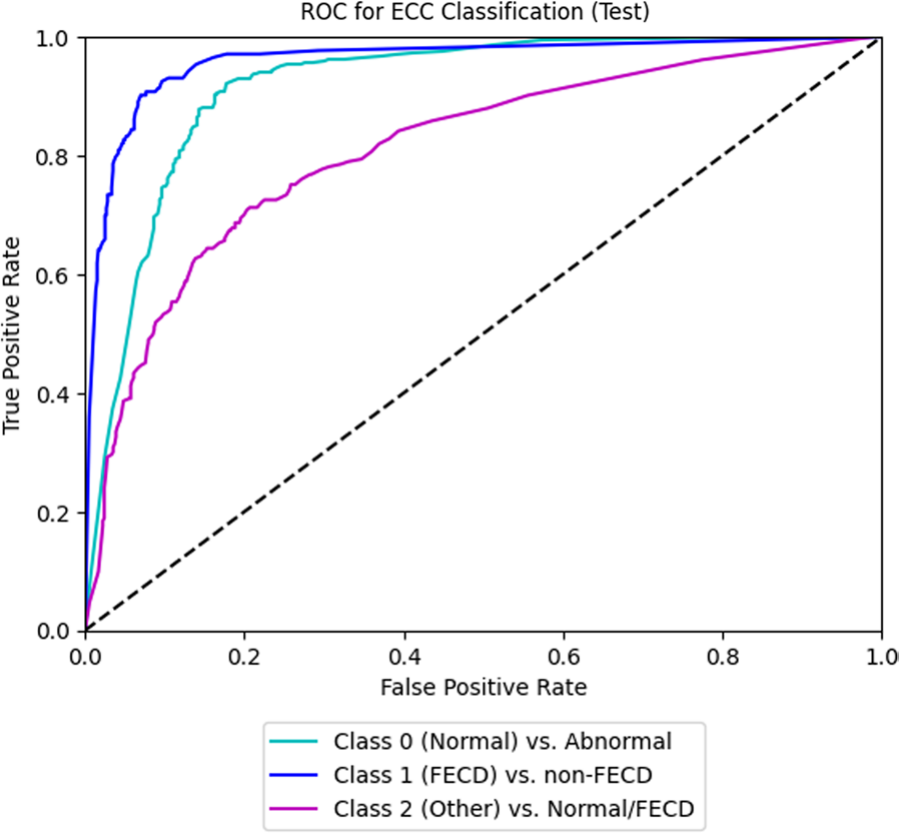
Receiver operating characteristic (ROC) curves for detection of normal *vs*. abnormal (Class 0), Fuchs endothelial corneal dystrophy (FECD) *vs*. non-FECD (Class 1) and other *vs.* normal/FECD (Class 2), based on specular imaging in the internal validation dataset. ECC, endothelial cell count

**Fig. 5:**
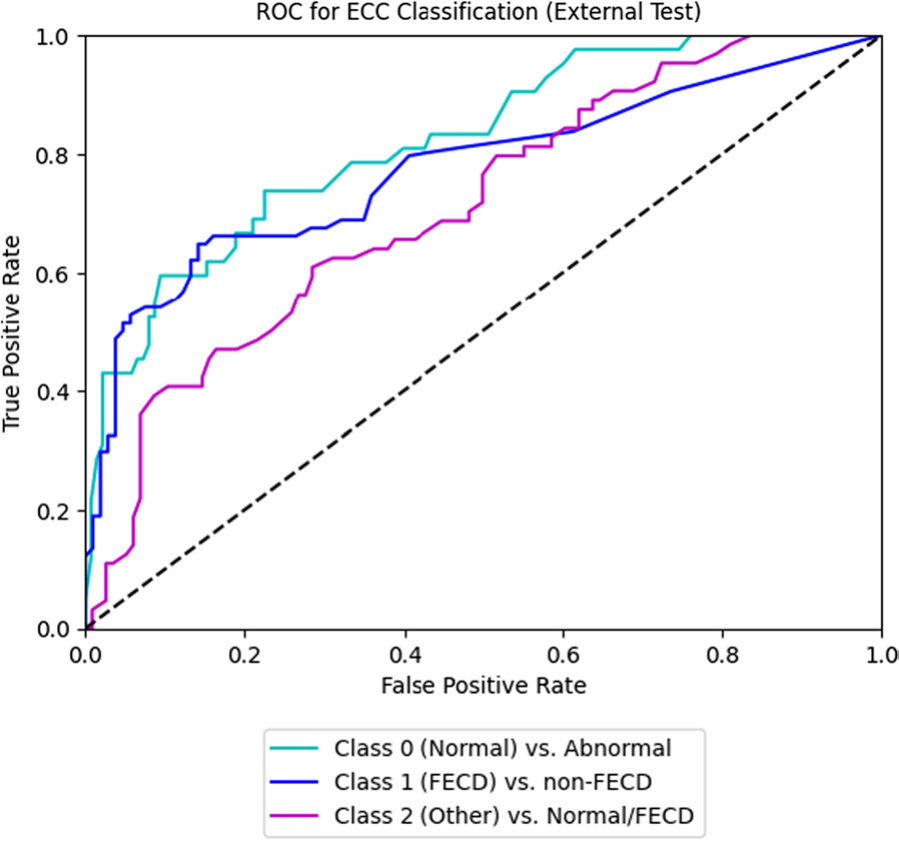
Receiver operating characteristic (ROC) curves for detection of normal vs. abnormal (Class 0), Fuchs endothelial corneal dystrophy (FECD) vs. non-FECD (Class 1) and other vs. normal/FECD (Class 2), based on specular imaging in the external validation dataset. ECC, endothelial cell count

In differentiating between FECD and other diagnoses, the internal validation set demonstrated an AUC of 0.96 (95% CI: 0.94–0.98), with a sensitivity of 0.91 (95% CI: 0.87–0.96) and specificity of 0.91 (95% CI: 0.90–0.94) (Fig. 4). The external validation set demonstrated AUC of 0.77 (95% CI: 0.69–0.76) with a sensitivity of 0.69 (95% CI: 0.62–0.72) and specificity of 0.68 (95% CI: 0.61–0.74) (Fig. 5). We compared the performance of our AI with that of an experienced cornea specialist (E.W.) in grading the SM images for FECD *vs.* other non-FECD diagnoses and found equivalent or superior results of the AI compared to the human grader. The human grader achieved a sensitivity of 0.33 and specificity of 0.96 in differentiating abnormal *vs.* normal SM images, and FECD from the other diagnoses with a sensitivity of 0.89 and specificity of 0.94.

For the second DL model, to identify widefield SM images with ECD > 1000 cells/mm^2^ in eyes with FECD, a sensitivity and specificity of 0.79 (95% CI: 0.70–0.86) and 0.78 (95% CI: 0.74–0.81) were achieved respectively, with an AUC of 0.88 (95% CI: 0.78–0.85).

**Fig. 6:**
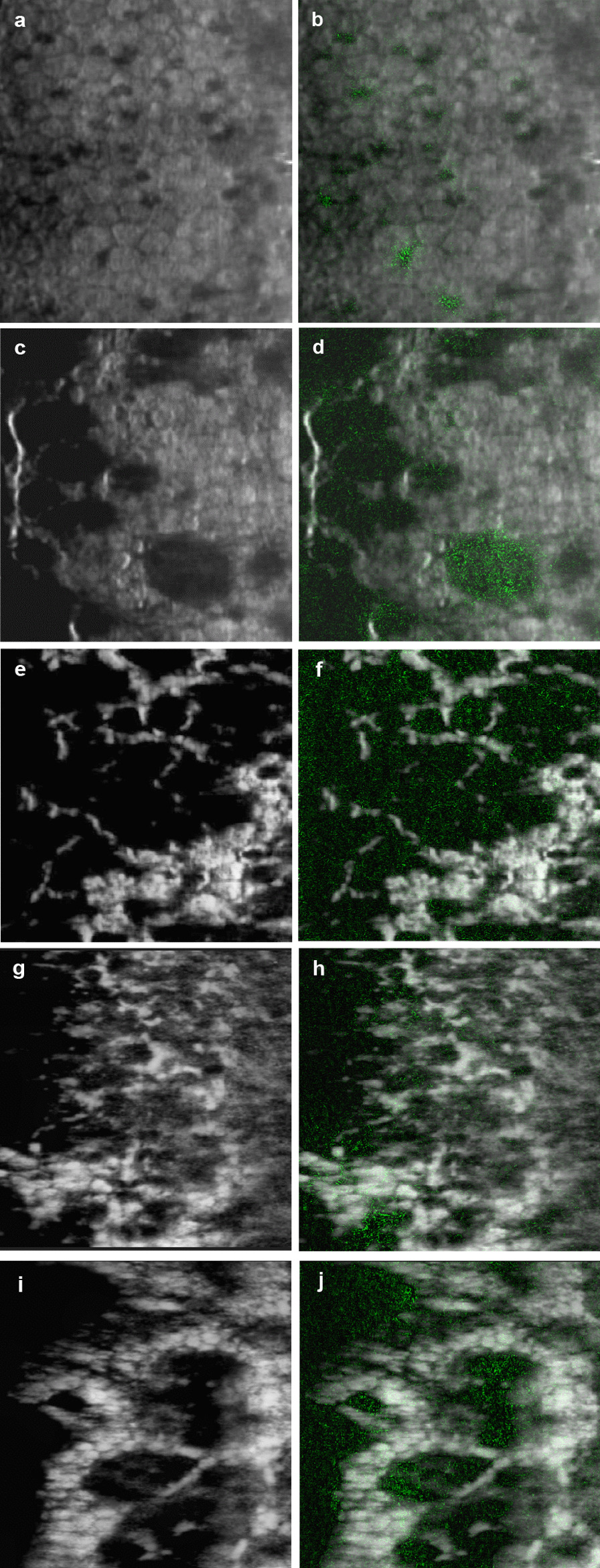
Saliency maps highlighting regions which the deep learning algorithm evaluated. **a,** **b** Uveitis with pigments seen. **c**, **d** Central specular microscopy (SM) images of Fuchs endothelial corneal dystrophy (FECD) with non-confluent guttae seen. **e**, **f** Central SM images of FECD with confluent guttae seen. **g**, **h** Paracentral SM images of FECD with guttae seen. **i**, **j** Peripheral SM images of FECD with guttae seen

Saliency maps highlighted regions within the central, paracentral, and peripheral SM images which the DL model likely focused on when identifying FECD amongst other diagnoses (Fig. 6a–j**)**. Generally, the highlighted regions corresponded well within guttae for FECD. Building on these illustrations, these clinically informative saliency maps could be incorporated as part of the screening algorithm. Normal images did not show any standout features on the saliency maps.

## Discussion

Here, we have described the design and validation of a fully automated DL model that could reliably detect specular images of FECD from non-FECD eyes with a sensitivity of 91% and specificity of 91%. The algorithm showed equivalent or superior performance to that of a trained experienced human cornea specialist. In addition, our DL model could distinguish peripheral specular images with an ECD > 1000 cells/mm^2^ from those with ECD ≤ 1000 cells/mm^2^ in FECD eyes, with a sensitivity and specificity of close to 80%. To the best of our knowledge, our study is the first to use a DL approach to automatically diagnose FECD based on SM images and identify widefield SM images with healthy peripheral endothelial reserves that could aid corneal surgeons in identifying suitable eyes for earlier therapeutic measures such as DSO.

Earlier studies have developed DL systems for segmenting corneal endothelial cells and deriving the morphometric parameters, using either specular microscopy [11–18] or in vivo confocal microscopy [19, 23] based on datasets comprising of either normal or FECD eyes. Unlike our study, these were either limited by smaller sample sizes, or did not pursue automated screening for abnormal SM images or FECD. Furthermore, we utilised ‘real-world’ SM images of various cornea endothelial abnormalities to train the DL model. The more densely pixelated regions were congruent with guttae (Fig. 5), confirming that the algorithm was able to detect FECD based on clinically appropriate features of the disease [20]. In addition, our DL model could identify peripheral SM images with ECD > 1000 cells/mm^2^ in FECD eyes. In the early stages of FECD, changes in endothelial morphology are observed centrally, before manifesting in the peripheries [21, 22]. Due to significant regional variations in the distribution of guttae and endothelial cell changes, the widefield SM could provide more information apart from central specular imaging in FECD eyes. Thus, our DL model could potentially assist in screening for suitable FECD eyes that might benefit from earlier treatments such as DSO with topical Rho-associated protein kinase inhibitor (ROCK-I) application. Future studies with larger data sets from various patient cohorts in real healthcare settings are needed to evaluate and determine the utility of these algorithms for patient selection and treatment response from such therapies. It is important to also note that as the retrospective datasets have undergone extensive filtering and cleaning, they were likely less representative of the real-world practice and hence may yield suboptimal performances when applied to clinical practice. The training methodology included early stopping on the accuracy of an independent validation set with all classes sampled to the same number of images, as a best practice to mitigate oversampling. Despite this, performance on our internal dataset remains higher than that on the external datasets, which may be attributed to the external datasets not obeying the same data distribution as the internal dataset, possibly due to subject/imaging differences. Therefore, performance on the external datasets would reflect the performance of the DL algorithm under more realistic conditions.

Despite the promising results from our pilot study, we recognise the limitations of our early results. First, our dataset is derived from a single center, which limits the generalisability of our results. Internal and external validation with a trained cornea specialist was hence performed to evaluate the clinical utility of our algorithm, which demonstrated that our algorithm performed equivalent or superior to the trained human grader. Larger studies would still be needed to evaluate its real-world performance. In SM images with corneal edema, the specular reflection is affected, which prohibits the visualisation of the corneal endothelium and precludes accurate analysis by the algorithm. Hence, we excluded SM images of eyes with significant corneal edema in our datasets, which helped to ensure a high-quality training set to ensure optimal performance of our algorithm. Moreover, for our training and validation datasets, the eyes with FECD were not stratified according to disease severity as we also did not collect data on the exact severity grading of FECD for each eye according to the Kracher grading, or specify which eye had confluent or non-confluent guttae. Hence, our test sets may have limited cases for each subtype of FECD. While our algorithm performed well in screening abnormal SM images, it needs to be further finetuned to reduce misdiagnoses or unnecessary referrals. Images that were misclassified by the first algorithm i.e., 55/775 (7.1%) considered ‘normal’ were from old pigments or keratic precipitates and had fairly normal images with borderline SM parameters. On the other hand, 65/775 (8.4%) were considered ‘abnormal’ due to imaging artifacts. Therefore, additional refinement of the algorithm will be needed to enhance differentiation between artefacts and pigments from guttae in FECD eyes. Despite their potential to support clinical practice, the generalisability of these AI models to large-scale populations remains uncertain and require validation through large randomised controlled trials to demonstrate their added clinical value before their widespread adoption.

## Conclusion

In conclusion, we describe the development of a DL model that could be used to detect FECD from specular microscopy, which requires further refinement and validation in other populations. A further DL technique could also detect eyes with central guttae with a healthy ECD in peripheral specular images in eyes with FECD. If validated, these algorithms could be a useful assistive device in the early detection, disease monitoring and patient selection for the treatment of corneal endothelial diseases such as Fuchs dystrophy. The findings of our study is the necessary first step in that process of development for future work.

## Data Availability

The data that support the findings of this study are available from the corresponding author upon reasonable request.
